# The anurans and squamates assemblage from Final Natufian Eynan (Ain Mallaha, Israel) with an emphasis on snake-human interactions

**DOI:** 10.1371/journal.pone.0247283

**Published:** 2021-02-25

**Authors:** Rebecca Biton, Salvador Bailon, Michal Birkenfeld, Anne Bridault, Hamoudi Khalaily, François R. Valla, Rivka Rabinovich

**Affiliations:** 1 Department of Bible, Archaeology and the Ancient Near East, Ben-Gurion University of the Negev, Beer-Sheva, Israel; 2 National Natural History Collections, The Hebrew University of Jerusalem, Jerusalem, Israel; 3 UMR 7209 Archéozoologie, Archéobotanique: Sociétés, pratiques et environnements, MNHN, CNRS, Paris, France; 4 UMR 7194 Histoire naturelle de l’Homme préhistorique, MNHN, UPVD, CNRS, Paris, France; 5 GIS Research Branch, Israel Antiquities Authority, Jerusalem, Israel; 6 CNRS, UMR 7041 ArScAn–Archéologies environnementales, MSH Mondes René Ginouvès, Nanterre, France; 7 Israel Antiquities Authority, Jerusalem, Israel; 8 National Natural History Collections, Institute of Earth Sciences, Institute of Archaeology, The Hebrew University of Jerusalem, Jerusalem, Israel; State Museum of Natural History, GERMANY

## Abstract

During the Natufian period, more than 12,000 years ago, Eynan (Ain Mallaha) was an important human settlement in the Hula Valley, Israel. This study concentrates on the anuran and squamate assemblage from the ultimate stage of the Natufian period at the site, the Final Natufian. Over five thousand bones assigned to at least sixteen taxa were studied from a sampled segment of the excavated open-air site. Relative species abundance, spatial distribution, taphonomic observations and ecological considerations all pointed to the conclusion that the inhabitants of Eynan intensively exploited three large “colubrine” snakes species: the Large Whip Snake (*Dolichophis jugularis*), the Eastern Montpellier Snake (*Malpolon insignitus*) and an Eastern Four-lined Ratsnake (*Elaphe* cf. *sauromates*). These snakes were the most desired and were intensively gathered, while other snakes and lizards could have been opportunistically collected when encountered. We raise questions about whether the large “colubrines” exploitation should be interpreted as additional evidence of increasing diet breadth. We suggest challenging this line of reasoning and offer possible alternative motives.

## Introduction

Snakes and humans have continuously interacted in areas where both taxa were present since the dawn of humanity. The relationship between snakes and humans was probably always complicated, combining fear with exploitation and worship. Fear of snakes was most probably evoked by specific physical and behavioral characteristics such as their silent slithering, ambush attacks, swallowing their prey whole, their venom, scaly skin, a forked tongue and a pointed head. Although most snakes will avoid contact with humans if possible, they are often considered harmful and therefore are abused or hunted. Nevertheless, at least some of the snake’s characteristics have given rise to ophiolatry (snake worship). For example, the ability to shed their skin and still survive is a reason snakes are thought to possess regenerative powers and also one of the reasons that the snake is a symbol of medicine [1, 2].

Snakes are abundant throughout the warm Middle East, and nowadays, more than 40 species are present in Israel [3]. Israel is rich in prehistoric sites [4], and at most of them snake bones appear alongside anthropogenic artifacts, probably because snakes often use caves for overwintering and seldom do not survive. Thus, it seems as if humans and snakes were sharing the caves and shelters simultaneously, when in fact their activities were probably successive. Another possibility is that the snake-remains were deposited by non-human predators. As a result, snake bones recovered at prehistoric sites in Israel are often from an uncertain origin and not included in the dietary analysis [5, 6], and the possibility that large snakes, especially large “colubrines” (see our definition of “colubrine” in the data collection section) were human prey is left open [5]. However, occasionally snakes vertebrae that were retrieved from Upper Paleolithic contexts [7], but mostly from assemblages dated towards the end of the Paleolithic periods, were reported as being part of the human diet and classified as food refuse [8–12].

This study aimed to investigate thoroughly the anuran (frogs and toads) and squamate (lizards and snakes) assemblage from the Final Natufian (Layer Ib) open-air site of Eynan (Ain Mallaha). Based on results that indicate the exploitation of snakes, we further attempted to decipher the relationship between snakes in the vicinity of the site and Eynan’s inhabitants.

The archaeological site of Eynan (Ain Mallaha) is located in the vicinity of a perennial spring in western part of the Hula Valley, North Israel. Several human occupations dated to different prehistoric periods were exposed there [13, 14]. The site was occupied at least intermittently for several millennia. During the Natufian period, it was one of the most important human settlements in Israel, as attested by its large size and the duration of its occupation, which started during the Early Natufian, approximately dated to 14,326 ± 266 cal BP. This study concentrates on the ultimate stage of the Natufian period at the site, the Final Natufian (from ca. 12,466 ± 179 to 11,895 ± 141 cal BP) [14]. The pattern of human occupation at that time is not yet fully understood in detail. Altogether the data at hand suggest at least repeated long stays of people. This is underlined by intensive activities, including the digging of semi-subterranean oval buildings, burying the dead, tool making, artwork, and more [14], as well as intensive exploitation of every ecological niche available near the site, and game procurement throughout the year, including fish, birds and mammals [15–22]. Each building went through several phases of occupation, some of them involving changes in function (dwellings versus other activities), as shown by reorganizations of postholes and hearths inside the buildings. Moreover, it seems that some species, such as the house mouse and possibly the fox, took advantage of the creation of new microenvironments by long-term human presence [18, 23]. On the other hand, there are indications that individual buildings were temporarily abandoned [24].

## Materials and methods

### Material origins and recovery methods

All the material studied and reported herein originated in Eynan’s Final Natufian deposits (Layer Ib) excavated by F. R. Valla and H. Khalaily between 1996–2005 (Israel Antiquities Authority permit numbers: G-81/1996, G-53/1997, G-77/1998, G-60/1999, G-51/2000, G-62/2001, G-44/2003, G-20/2004 and G-51/2005). Generally speaking, the excavation was conducted using a unit system of a quarter square meter (0.25 m^2^), 5 cm deep. This system was adapted according to the findings. The undifferentiated top of Layer Ib was excavated using a 1m^2^ grid, shifting to the quarter square meter grid inside and outside structures, as soon as they were identified. The fill of each structure was isolated, introducing ‘natural’ boundaries instead of the artificial limits established by the grid. Nevertheless, in each structure, the artificial grid subdivisions were respected, to allow spatial distribution of the small finds.

The thickness of the units was also adapted to the stratigraphy, resulting in units much thinner than 5 cm, especially when in search of ‘floors’ and surfaces. As a rule, during excavation only exceptional pieces were spatially recorded in the undifferentiated upper part of Layer Ib, which was crowded with small limestone blocks. Inside the structures, all item larger than 5 cm (bones, stones, lithic artifacts and other finds) were mapped. Sediments were then wet-sieved using a 1–2 mm mesh and further sorted in the laboratory.

**Fig 1:**
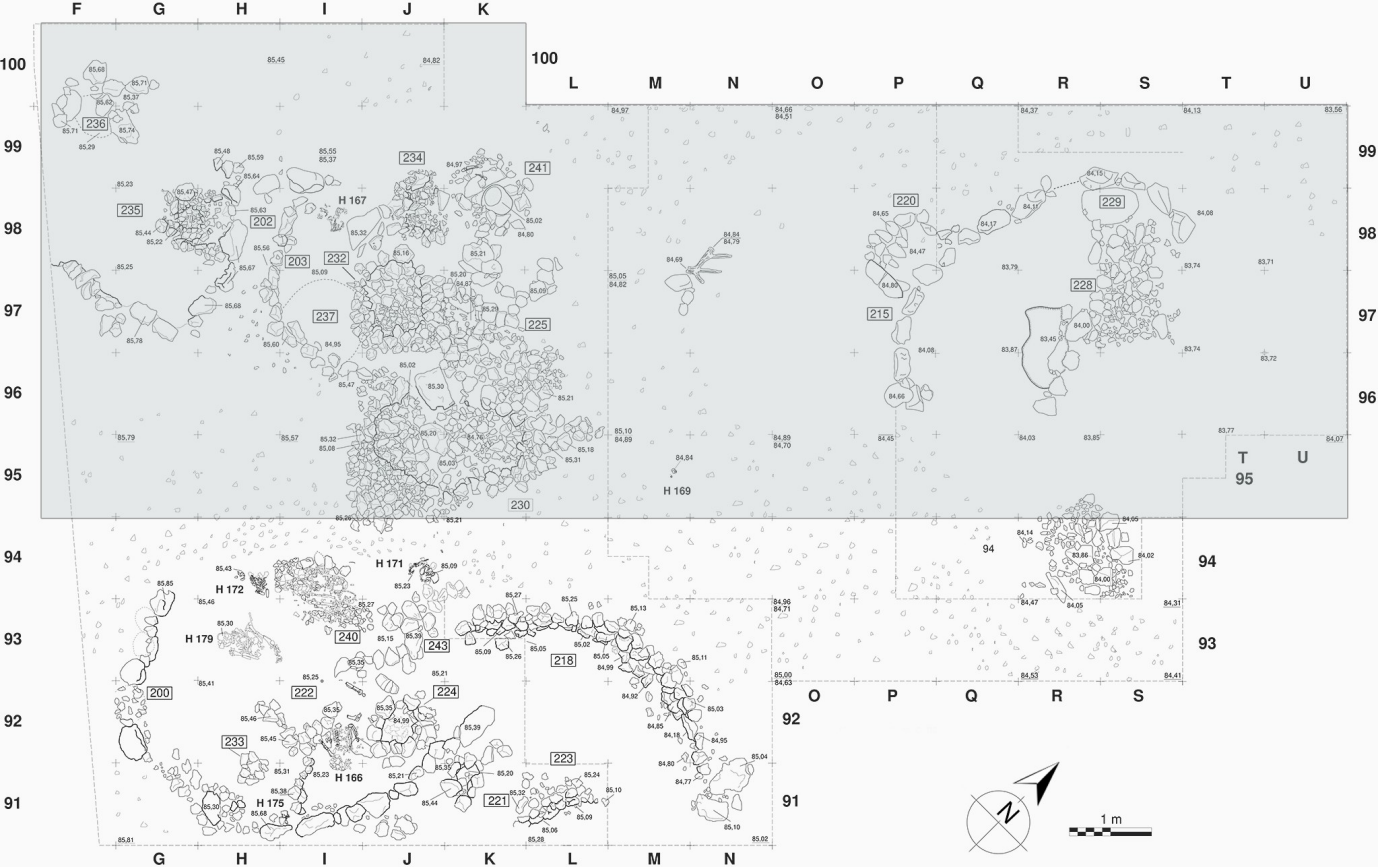
Final Natufian (Layer Ib) excavation map. Map of the area exposed at Eynan (Ain Mallaha) during the 1996–2005 excavation campaigns, with an indication of the area sampled for the current study. Figure designed by F. R. Valla based on data from fieldwork.

Due to the large volume of material retrieved from the excavation area, we chose to study and publish herein the material originating from approximately half the Final Natufian surface exposed (squares F-U/95-100) (Fig 1).

### Data collection

Each anuran and squamate bone retrieved received an individual number, and all variables regarding the definition of taxa, element, completeness, and surface modifications were recorded in a database. All the remains are now housed at the National Natural History Collections at the Hebrew University of Jerusalem, Israel (NNHC-HUJ), according to taxa.

Body parts were defined first. The snakes vertebral column was divided into four regions: cervical, trunk, caudal and cloacal following Szyndlar [25]. Then, body parts were assigned to the lowest taxonomic rank possible. In order to identify the bones, we used the comparative collections of the National Natural History Collections at The Hebrew University of Jerusalem, Israel (NNHC-HUJ) and the Muséum national d’Histoire naturelle Paris, France (MNHN). The taxonomic nomenclature follows two periodically updated websites: AmphibiaWeb (http://amphibiaweb.org/) for anurans and The Reptile Database (http://www.reptiledatabase.org/) for the squamates.

“Colubrines” are defined here following Szyndlar [26]. The term was coined by paleontologists following the division of Colubridae (s. l.) to two informal subfamilies, “colubrine” and “natricine”, based on the absence or presence of hypapophyses on the trunk vertebrae (post cervical), respectively. The informal term “colubrine” used herein therefore includes Psammophiidae species (for example *Malpolon insignitus* and *Psammophis* cf. *schokari* identified at Eynan), diverging from systematic researches [27, 28].

The Number of Identified Specimens (NISP) was recorded for all taxa. The Minimum Number of Individuals (MNI) calculations were based on the highest count of elements standardized by the bone side for paired elements, taking into account completeness. However, determining the minimum number of individuals (MNI) for snakes was not an easy task, mainly because the majority of the remains were vertebrae, and a single snake has hundreds of them. Besides vertebrae being numerous in comparison to other elements, their robustness also ensures their better preservation. Snakes skull-elements that are more suitable for assessing MNI were extremely rare in the material studied because they are also less robust than vertebrae and therefore have a poor rate of preservation. In contrast to the snakes, the anuran ilia and lizard jaws that are robust elements are also appropriate for taxonomic assignment. Assessing snakes MNI is therefore not as easy as assessing anurans or lizards MNI.

An attempt was made to assess snakes MNI according to the number of trunk vertebrae that are posterior to the cervical vertebrae and anterior to the cloacal and caudal vertebrae. Trunk vertebrae were chosen since they bear the most distinctive characteristics for identification and are the preeminent and sometimes the only vertebrae that allow taxonomic assignment to species level. Based on multiple large “colubrines” specimens stored at the osteological comparative collections at the NNHC-HUJ, the snake species identified at Eynan have 122 to 167 trunk vertebrae per specimen, (S1 Table). Therefore, the minimum number of individuals (MNI) was assessed by dividing the number of trunk vertebrae identified to a specific taxon at Eynan by 167, the highest number of trunk vertebrae possible according to the comparative collections specimens. As a result, our snake MNI is very conservative and is, without a doubt, massively lower than expected.

### Taphonomy

Each bone was examined under a light microscope at 10× to 40× magnification for surface modifications caused by abiotic and biotic processes, such as carnivores, raptors, and human predation [29]. Surface modification in anurans was inspected following literature [30–32]. Actualistic taphonomic studies directed toward raptors preying on anurans and squamates were also consulted [12, 33–36]. For the snake vertebrae alterations, we followed the five digestion categories as published and well-illustrated by Lebreton et al. [36] that were available to us for comparison. However, we merely referred to the categories for measuring the degree and location of modifications on vertebrae, without deducing if they were produced by digestion or corrosion by other agents (referred to as erosion by Lev et al. [12]). The reason is that, although some differences in the types of modification produced by digestion and corrosion were reported (e.g. regular edges versus irregular edges [12]), the distinction between them is not always straightforward [29]. The categories that were followed (after Lebreton et al. [36]) are: (0), non digested (here not affected); (1) at least one of the diapophysis or parapophysis affected; (2) the addition of prezygapophyseal processes to the previous areas affected; (3) the addition of the condyle; (4) the addition of lamellar bone loss (Figs 8 and 9 in [36]).

### Spatial distribution

The spatial distribution of all bones was analyzed using ESRI ArcMap 10.7.1. Items were plotted according to their grid-square location. In order to resolve any variations resulting from differential excavation depth in different grid squares, the sum of all items recovered from a single square was first divided by the depth of excavation in that square (in cm). The result represented the average sum of bones per 1 cm in each excavated square, on which all analyses were performed. The reasoning behind this was that during excavation, some areas of interest were excavated deeper than others. This is true especially for the semi-subterranean buildings that were dug by Eynan’s inhabitants who took advantage of a slope overlooking the spring and typically formed the boundary upslope by a semi-circular wall. Moreover, some buildings were dug by the Natufians into a surface more or less equal to the top of Layer Ib, while previous structures in the same layer were covered by some 20 cm of sediment, for example, the wall of building 215 [16].

First, a simple distribution map by grid-square was produced for each tested category. Counts were presented using the Natural Jenks, based on Jenks’ optimization method [37], which seeks to reduce variance within groups and maximize the variance between them. The Natural Jenks method partitions data into different classes using breaks or gaps that naturally exist in the data. Thus, it calculates the groupings of data values based on natural groups in the data distribution. Second, the Getis-Ord Gi* statistic (Hot-Spots Analysis) was used to evaluate the distribution of the bones. The Getis-Ord Gi* method assesses local association among neighboring data, by comparing local (weighted) averages to global averages and looking for statistically significant clusters of high and low values [38].

## Results

**Fig 2:**
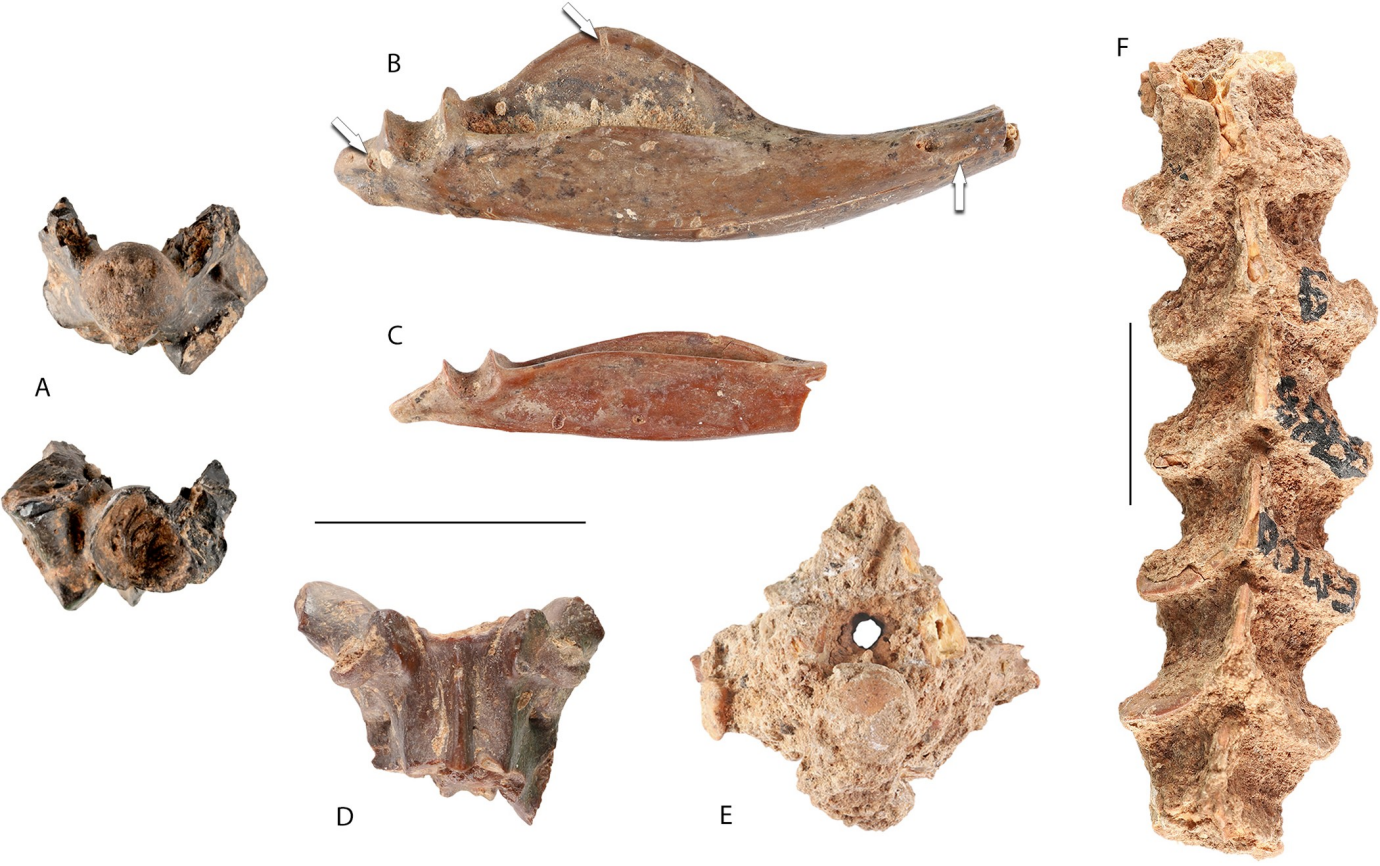
Taphonomic observations on snake bones from Final Natufian Eynan (Ain Mallaha). All scale bars equal 10 mm. Arrows point at modifications that were tentatively identified as root etching. (A) EM-25363 posterior and anterior views of a large “colubrine” vertebra without neural arch; (B) EM-7332, lateral view of a *Malpolon insignitus* right compound bone fragment; (C) EM-24746, lateral view of a *Dolichophis jugularis* right compound bone fragment; (D) EM-25913, ventral view of a large “colubrine” vertebra with broken posterior portion; (E) EM-13085, anterior view of an encrusted large “colubrine” vertebra; (F) EM-14459, dorsal view of six encrusted *Malpolon insignitus* articulated vertebrae. All photographs by Assaf Uzan.

The anuran, lizard and snake remains studied herein include 5364 bones, representing at least 16 taxa (Tables 1 and 2) retrieved from approximately half of the area excavated for the Final Natufian Layer Ib at Eynan (Fig 1). The preservation of the remains is overall poor. Most of the anuran and squamate bones have various surface modifications that were tentatively identified as root etching (Fig 2B) and linear marks produced by trampling, alongside rare marks of rodent scratching/chewing. Fragmentation as a result of post-depositional processes such as trampling and soil compaction, was already reported for other taxonomic groups [18]. Anurans and squamates were affected by these processes as well.

**Table 1:** Anurans and lizards taxa from Final Natufian Eynan (Ain Mallaha).

	Anuran				Lizard					
	*Latonia nigriventer*	*Bufotes* sp.	*Pelophylax* cf. *bedriagae*	Anura indet.	*Stellagama* cf. *stellio*	Chamaeleonidae	*Eumeces* cf. *schneideri*	Lacertidae	*Pseudopus apodus*	Lizard indet.
NISP	10	24	65	36	88	2	33	8	58	56
MNI	2	2	13		18	1	11	2	1	

Number of Identified Specimens (NISP) and Minimum Number of Individuals (MNI).

**Table 2:** Snakes taxa from Final Natufian Eynan (Ain Mallaha).

	*Dolichophis jugularis*	*Malpolon insignitus*	*Elaphe* cf. *sauromates*	Large “colubrine” indet.	*Hemorrhois nummifer*	*Psammophis* cf. *schokari*	“Colubrine” indet.	*Natrix* sp.	*Eryx* sp.	cf. *Daboia palaestinae*	Snake indet.
NISP	450	277	166	1537	28	11	268	28	55	8	2156
MNI	4	2	1	10	1	1	2	1	1	1	

Number of Identified Specimens (NISP) and Minimum Number of Individuals (MNI).

The most obvious and straightforward evidence of human consumption of small animals, including anurans and squamates, are coprolites. Examples of human coprolites containing small animals are rare [39]. Still, some were recovered at several archaeological sites with evidence to the consumption of snakes [40, 41], anurans [42], and lizards [43]. Unfortunately, human coprolites were not found at Eynan. Other signatures of human exploitation are less distinct, mainly due to the varied modes of capture, preparation, and discard that could influence skeletal representation and bone modifications. At Final Natufian Eynan, it seems that the post-depositional processes were so intense that they blurred any previous marks on the bones. Cutmarks are signs of human exploitations, but they are very rare on small animals that hardly ever require the use of sharp tools to process them. All bones at Eynan were inspected for cutmarks, but none was detected.

An additional topic we wanted to tackle was the possible relationship of the graves and the dead to anurans, squamates, and most specifically to snakes. At Final Natufian Eynan, dead were buried without grave goods or ornaments, inside dwellings, and alongside different structures. Some burials were dug into the sediment, and on one occasion, it seems that the deceased was put on a floor and, after a while, covered with sediments [24]. We could not find any correlation between the burials and specific anuran or squamates taxa recovered (S2 Table). Overall it seems that the taxa in the graves are the same as those scattered throughout the site.

### Anurans

The anuran remains include 135 bones. The most common element is the ilium bone that was used to calculate the MNI. A minimum of 17 individuals was calculated, representing at least three taxa of frogs and toads (Table 1). The most abundant taxa is the Levant Water Frog (*Pelophylax* cf. *bedriagae*). Almost none of the anuran bones are complete, and 4% of the bones were heavily digested, maybe by a mammalian predator. A total of only six anuran bones with burning marks was recorded, representing 4.4% of the anuran assemblage.

**Fig 3:**
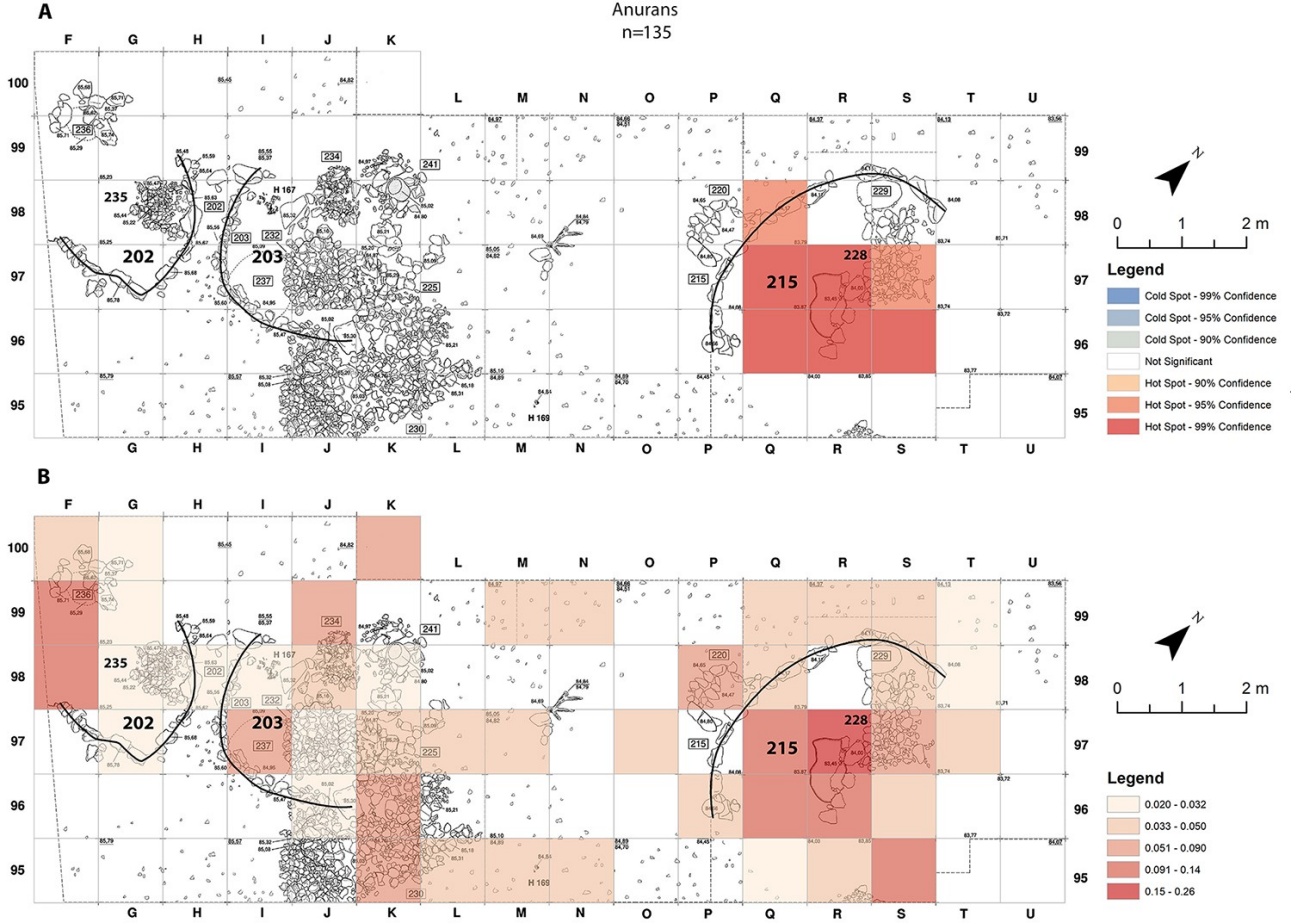
Anuran-bones distribution maps from Final Natufian Eynan (Ain Mallaha) with indication of the major buildings (202, 203 and 215). (A) “Hot-Spots Analysis Map” using Getis-Ord Gi statistic. (B) Natural Jenks distribution map based on the average sum of anuran bones per 1 cm depth for each grid-square. The maps were created based on the modification of Eynan’s excavation map (Fig 1) and the bones grid-square location (https://doi.org/10.17026/dans-2c3-mn7q) using ESRI ArcMap 10.7.1.

The spatial distribution of each taxon within the excavated area was compared to all the other taxa studied, with reference to the many buildings, structures and graves within the selected area. The spatial distribution of the anurans is mainly restricted to one area in squares Q-R-S/97-98, in the north-eastern part of the excavation, as can be seen from the very high confidence hot-spots in building 215 (Fig 3A). An additional possible concentration can be spotted on the simple distribution map by grid-square in squares F/98-99 in the western corner of the excavation (Fig 3B). A look at the vertical distribution of the items suggests a correlation in this area with the fill around hearth 235 [17]. Interestingly, area Q-R-S/96-97-98 is also the place where another large hearth (228 in building 215) was excavated [44]. However, as mentioned previously, anurans have low percentage of burning marks.

### Lizards

The lizard remains consist of 245 bones representing 33 different lizards (MNI) identified to five taxa of agamids, chamaeleons, skinks, anguids and true lizards (Table 1). The most abundant lizard (NISP and MNI) is the Roughtail Rock Agama (*Stellagama* cf. *stellio*). The dentary was the most abundant element that enabled calculating the MNI, for all taxa except for the European Glass Lizard (*Pseudopus apodus*). *P*. *apodus* has an MNI of a single animal, although the NISP is high relative to other lizards (NISP 58). Most of the *P*. *apodus* remains (NISP 45) are osteoderms that could easily belong to one animal, followed by vertebrae (NISP 11) and only two dentaries (one right and one left). This phenomenon of many *P*. *apodus* remains belonging to a single animal was already reported at the Mousterian open-air site of Nahal Mahanayeem Outlet [45]. A single *P*. *apodus* has approximately 150 vertebrae and more than a thousand osteoderms, and therefore a very high number of remains can relate to only one lizard.

**Fig 4:**
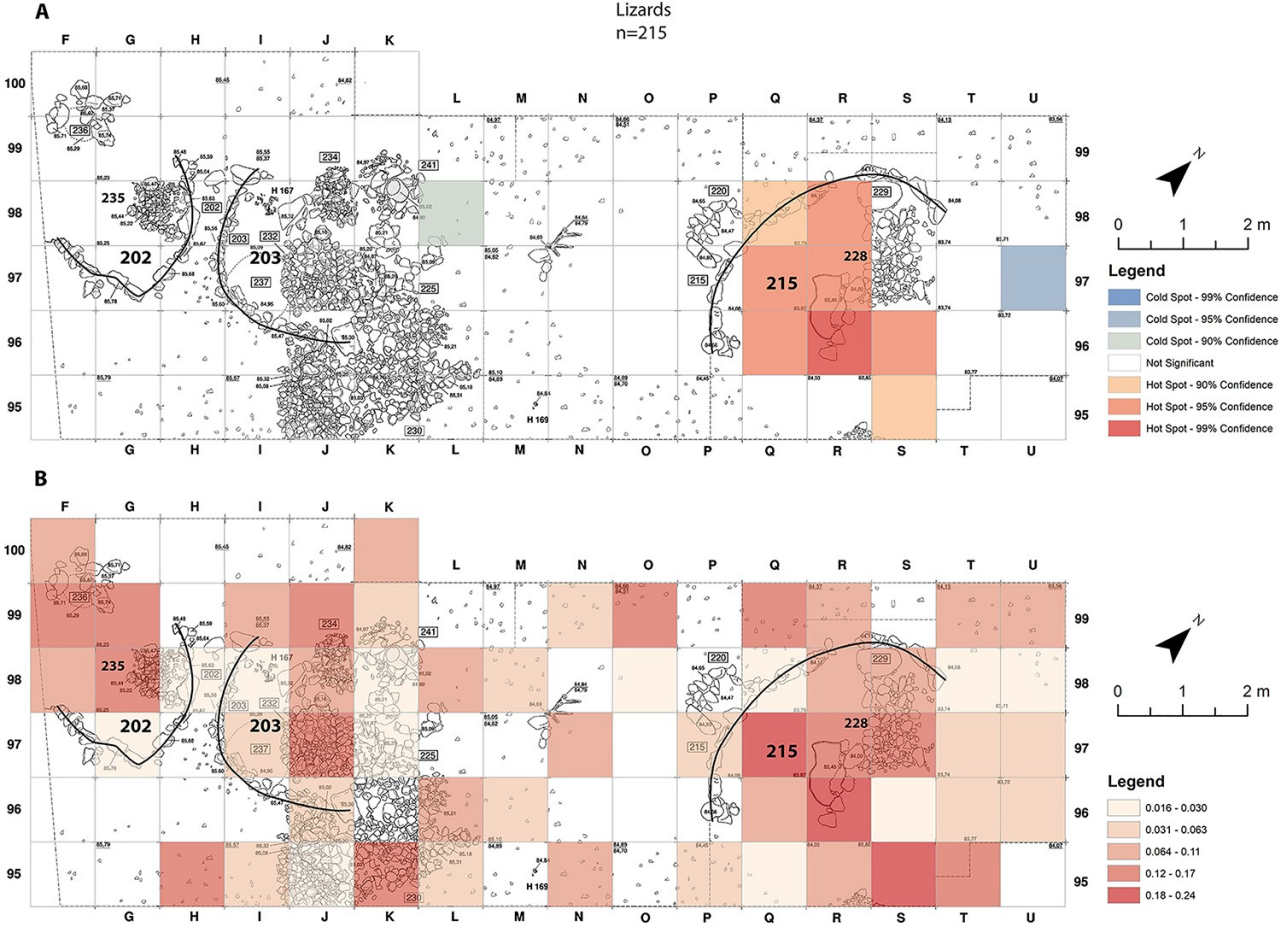
Lizard-bones distribution maps from Final Natufian Eynan (Ain Mallaha) with indication of the major buildings (202, 203 and 215). (A) “Hot-Spots Analysis Map” using Getis-Ord Gi statistic. (B) Natural Jenks distribution map based on the average sum of lizard bones per 1 cm depth for each grid-square. The maps were created based on the modification of Eynan’s excavation map (Fig 1) and the bones grid-square location (https://doi.org/10.17026/dans-2c3-mn7q) using ESRI ArcMap 10.7.1.

As expressed by discoloration of the bones, burning occurred on 12% of the lizard remains. The spatial distribution of the lizards is similar to the anurans. It is restricted to squares Q-R /96-97-98 and S/95-96, in the north-eastern part of the excavation, in and adjacent to building 215 (Fig 4). If so, some connection was detected between the inner part of buildings and the anurans and lizards. Interestingly no such connection was detected with building 203, the only building in the study-area that was interpreted as a dwelling, at least for part of its lifetime [46].

### Snakes

Snake bones are by far the most abundant squamate remains—4984 bones overall. The minimum number of snakes recovered (MNI) is 24, but this number is a very low estimation, and we suspect that the actual number is considerably higher (see materials and methods section for snake MNI calculation; Table 2). Burning marks were recorded on 29.25% of the snake bones. Abrasions on vertebrae are localized and were detected on protruding parts of the vertebrae, mostly on the prezygapophyses.

The snake assemblage is divided into several subgroups to facilitate the understanding of this varied assemblage: unidentified snakes, large “colubrines”, and rare snakes. Each group will be treated here separately since lumping them together, in our view, hides essential details.

#### Unidentified snakes

**Fig 5:**
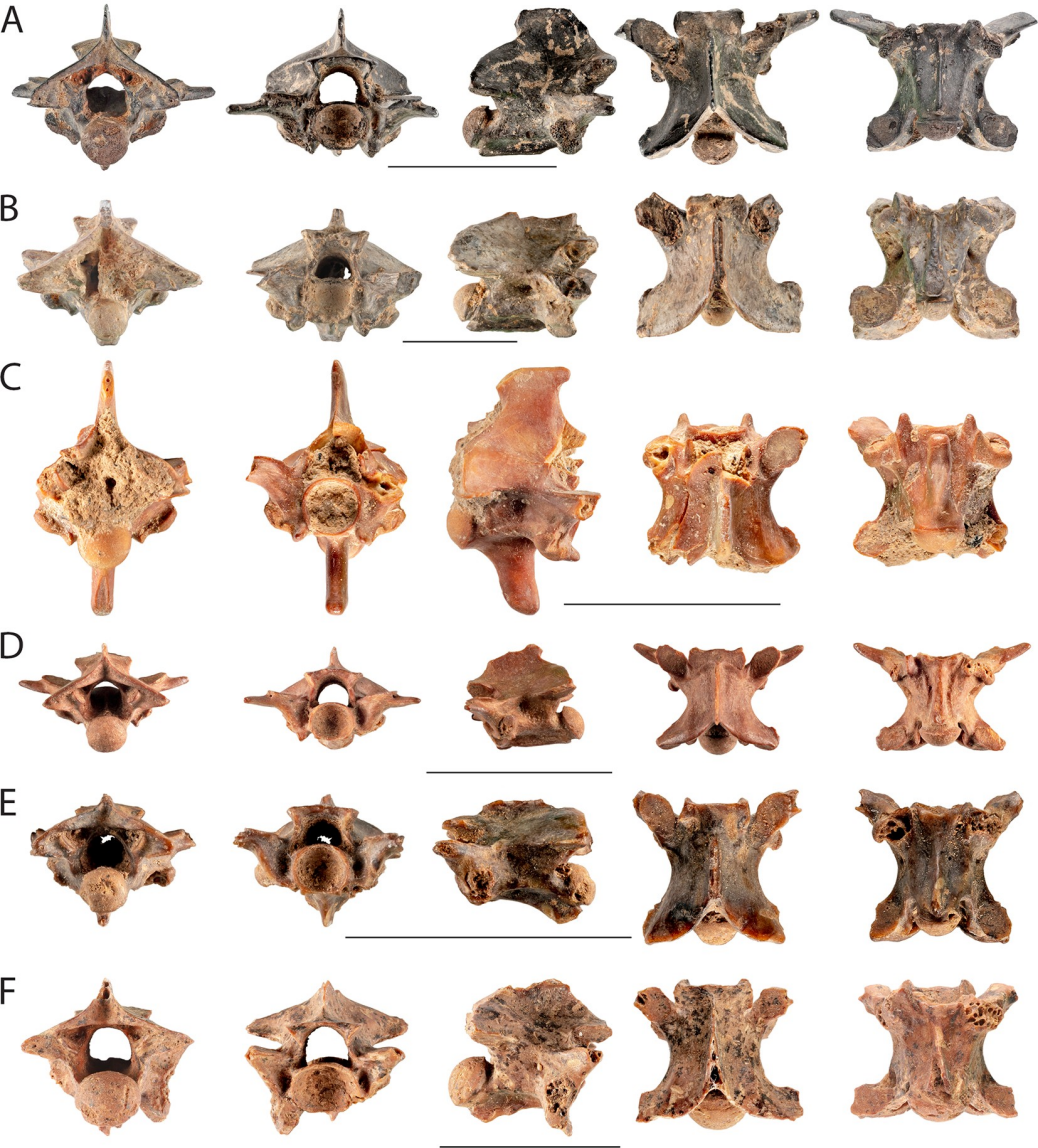
Snakes vertebrae from Final Natufian Eynan (Ain Mallaha). A-F posterior, anterior, lateral, dorsal, and ventral views. All scale bars equal 10 mm. (A) EM-20783, *Dolichophis jugularis* trunk vertebra; (B) EM-14500, *Malpolon insignitus* trunk vertebra; (C) EM-673, *Elaphe* cf. *sauromates* cervical vertebra; (D) EM-25035, *Hemorrhois nummifer* trunk vertebra; (E) EM-21915, *Natrix* sp. vertebra; (F) EM-25578, cf. *Daboia palaestinae* vertebra. All photographs by Assaf Uzan.

Unidentified snake remains are very abundant, second only to large “colubrine” remains, and include 2156 bones, 229 of them snake ribs, which are challenging to identify taxonomically below family. The rest are vertebrae (NISP 1927), which lack apparent morphological traits, either because they are poorly preserved or because they originate from sections of the column that are difficult to identify taxonomically. These include 77 cervical vertebrae that were not further identified to species, with exception of the cervical vertebrae of *Elaphe* cf. *sauromates* that bear hypapophyses directed anteriorly (Fig 5C). Szyndlar [25, 47] referred to *Elaphe quatuorlineata* when describing this feature, but based on the geographic distribution of the fossils studied by him and on more recent studies, those remains presumably belong to *E*. *sauromates* s. l. (formerly considered a subspecies of *E*. *quatuorlineata*) [48].

Most cloacal and caudal vertebrae, 841 in total, are also of low taxonomical value for most snake species, although given the size of the vertebrae most of them seem to have originated from large “colubrine” snakes. Needless to say, an MNI was not calculated for the unidentified snakes.

#### Large “colubrines”

Most of the snake bones, 2430 in total, were assigned to large “colubrine” snakes (Table 2). Three species were identified within this group; all are snakes that can exceed 2 m long. A total of 450 bones were identified as Large Whip Snake *Dolichophis jugularis* (Fig 5A), 277 as Eastern Montpellier Snake *Malpolon insignitus* (Fig 5B), and 166 as Eastern Four-lined Ratsnake *Elaphe* cf. *sauromates* (Fig 5C). Identification was based on cranial elements and trunk vertebrae, while *Elaphe* cf. *sauromates* was identified also on the basis of cervical vertebrae. In addition a total of 1537 trunk vertebrae and 27 skull bones were assigned to large “colubrines” and presumably belonged to one of the three species identified. The assignment of trunk vertebrae to large “colubrines” was based on morphological traits, especially on hypapophyse reduced to the haemal keel, and on the length of the centrum that exceeds 5 mm for fully adult specimens [25]. Skull elements are typical and include: two septomaxilla (basiparasphenoid), nine compound bones (Fig 2B and 2C), four dentaries, three maxillae, a palatine, a premaxilla, a pterygoid and six quadrates.

The large “colubrine” remains represent an MNI of 17 snakes: four *D*. *jugularis*, two *M*. *insignitus*, one *E*. cf *sauromates* and ten unidentified large “colubrines”. The MNI was calculated using the trunk vertebrae NISP as explained in the materials and methods. The exception is the MNI of four *D*. *jugularis* that was calculated on the basis of skull elements and more specifically, six compound bones (Fig 2C), of which four were left ones.

Some of the large “colubrines” recovered at Eynan were extremely large, and according to our estimation reached a length of up to 2.5–3 meters. We based our estimation on the centrum length measurement of trunk vertebrae, compared with recent specimens with known body length. The centrum length of the largest trunk vertebra of the largest *D*. *jugularis* (S1 Table) in the osteological comparative collections housed at the National Natural History Collections at the Hebrew University of Jerusalem, Israel (NNHC-HUJ) measures 7.7 mm. This snake’s total body length had been 193 cm. The centrum of 29 trunk vertebrae of *D*. *jugularis* retrieved from Eynan was longer than 7.7 mm. The centrum length of *D*. *jugularis* largest trunk vertebra reached 8.4 mm. Two large *M*. *insignitus* specimens from the comparative collection had trunk vertebrae with centrum up to 8.3 mm long (S1 Table). A total of 48 *M*. *insignitus* trunk vertebrae from Eynan had a longer centrum, 16 of them longer than 9 mm, and the longest reached 10.3 mm. Moreover, 40 trunk vertebrae assigned to unidentified large “colubrine” snakes at Eynan had a centrum longer than 8.3 mm.

An important characteristic that is exclusive to the largest vertebrae of the large “colubrines” in the anurans and squamates assemblage, is the calcite encrustation of the surface (Fig 2E and 2F). All faunal remains originating from Layer Ib, including all mammals, birds, and Testudines are thickly encrusted if the fragments are large enough for the crust to form [15, 18]. The encrustation hindered identification of the remains to the lowest taxonomic level possible and masked surface modification and burning signs on the bones, and seldom allowed the recovery of large “colubrine” vertebrae in articulation (Fig 2F). Large “colubrine” vertebrae were always broken to some degree, with the projecting parts including the neural spine, hypapophyses, zygosphene, and prezygapophyses mostly affected.

A remarkable fragmentation pattern of large “colubrine” vertebrae was observed on 29% of the unidentified large “colubrines” and 19% of all large “colubrines” vertebrae. In each of the affected vertebrae, more than half is missing: the dorsal part, including all the neural arch, and at least one prezygapophyse (Fig 2A). Dorsal parts (neural arch) were probably further broken and crushed, and were therefore never recovered at the site. We further checked if this pattern was more abundant on vertebrae that have signs of fire discoloration since burning is reported as enhancing post-depositional breakage for snake vertebrae [12]. No correlation was found however, 36% of the "less than half vertebra" show signs of exposure to fire, a very similar degree to that of other large “colubrine” vertebrae. Another feature, which is scarce (1.5% of the large “colubrine” vertebrae) but worth mentioning, is the breaking and loss of the condyle without heavy signs of corrosion or digestion (Fig 2D and 2F).

We scored the intensity of corrosion/digestion as expressed by the exposure of the underlying bone, after Lebreton et al. [36]. A total of 1986 snake vertebrae without encrustation, of the large “colubrines” and rare snakes subgroups, were scored (S3 Table). The most abundant category when the entire snake assemblage was treated as a single entity was category 4, which is related to heavy modification. If we separate the snake vertebrae according to taxa however, the picture is different. Unsurprisingly the unidentifiable large “colubrines” and the unidentifiable “colubrines” groups were the ones that were mainly subject to heavy modifications (35.6% and 55.9% respectively), a circumstance that influenced our ability to identify them to species. Since the snake vertebrae that were heavily corroded (category 4) did not display heavy rounding or polishing (abrasion) of the vertebrae, it seems that non-digestive corrosion would be a better explanation for the majority of the scored taphonomic modifications.

The snake-remains with the most burning marks are the large “colubrines” with 32.9% (Figs 2A and 5A and 5B). Within the large “colubrines”, *M*. *insignitus* has burning marks on 39.7% of its remains, followed by *D*. *jugularis* with 35.33%, and only then the *E*. cf. *sauromates* with 18.6%. In order to try and understand whether large “colubrines” that show high percentages of burning were burnt while the flesh was still on the bones or not, we looked at the burning distribution pattern on the snakes: head, trunk (trunk vertebrae), and tail (cloacal and caudal vertebrae). If the burning marks were the result of intentional exposure of complete snakes to the fire, we would expect that cervical, cloacal and caudal vertebrae, as well as skull elements, will show higher percentages of burning since these parts have less flesh on the bones and are at the extremities of the snake. Our results revealed an uneven distribution of burning marks, but with the opposite pattern to that expected for roasting a complete snake. The trunk vertebrae, which are the middle part of the snake and covered with most of the flesh, have 33.34% of the burning marks; the cloacal and caudal vertebrae have a slightly lower value of 29.8%; cervical vertebrae 24%; and skull elements 18.5%. Moreover, ribs that are relatively more exteriorly positioned than vertebrae have the lowest level of burning, only 15.72%.

**Fig 6:**
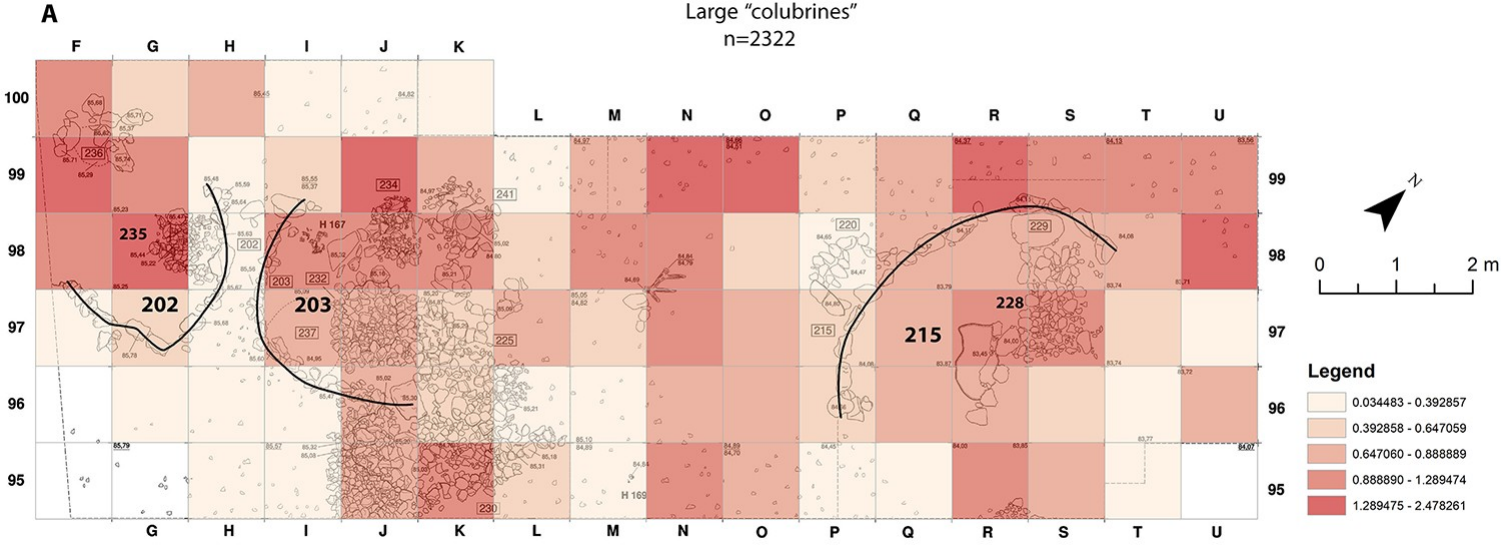
Large “colubrines” “Natural Jenks” distribution map from Final Natufian Eynan (Ain Mallaha) with indication of the major buildings (202, 203 and 215). Natural Jenks distribution map based on the average sum of large “colubrine” bones per 1 cm depth for each grid-square. All large “colubrines” taxa included: *Dolichophis jugularis*; *Malpolon insignitus*; *Elaphe* cf. *sauromates* and large “colubrines” unidentified to more specific taxa. The map was created based on the modification of Eynan’s excavation map (Fig 1) and the bones grid-square location (https://doi.org/10.17026/dans-2c3-mn7q) using ESRI ArcMap 10.7.1.

**Fig 7:**
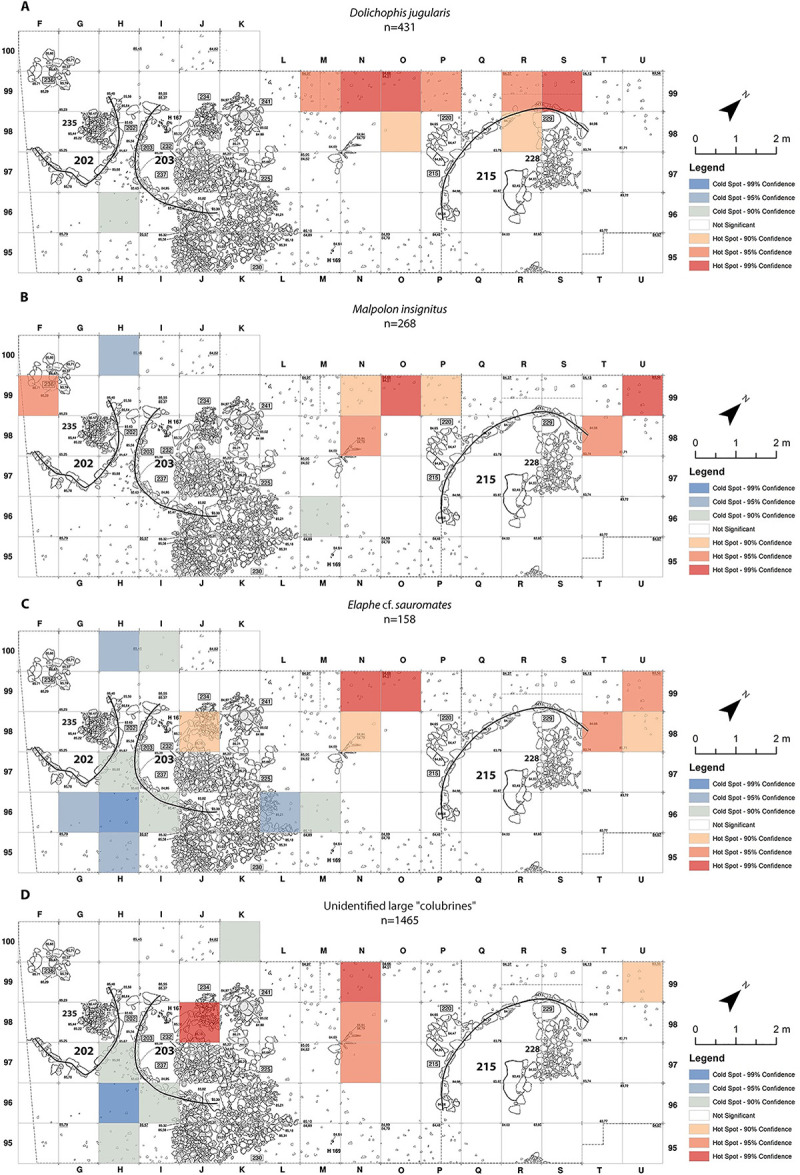
Large “colubrines” “Hot-Spots Analysis Map” using Getis-Ord Gi statistic from Final Natufian Eynan (Ain Mallaha) with indication of the major buildings (202, 203 and 215). (A) *Dolichophis jugularis*; (B) *Malpolon insignitus*; (C) *Elaphe* cf. *sauromates*; (D) large “colubrines” unidentified to more specific taxa. The maps were created based on the modification of Eynan’s excavation map (Fig 1) and the bones grid-square location (https://doi.org/10.17026/dans-2c3-mn7q) using ESRI ArcMap 10.7.1.

As opposed to the anurans and lizards spatial distribution-patterns (Figs 3 and 4), no connection was found between the inside of buildings and large “colubrine” remains. It is essential to emphasize that all large “colubrine” taxa were recovered all over the excavated area, including within buildings and dwellings, but their abundance varies (Fig 6). The hot-spots for the large “colubrines” are different from those for other taxa at the site. They are mainly in the north-west area of excavation, adjacent to the outside part of the arched wall of building 215 (Fig 7). Interestingly, in the area outside the wall of dwelling 203 cold-spots were identified, indicating an extremely low abundance of large “colubrines” in this area.

#### Rare snakes

This subgroup includes 134 snake bones of five taxa: *Hemorrhois nummifer*, *Psammophis* cf. *schokari*, *Natrix* sp., *Eryx* sp. and cf. *Daboia palaestinae*, with less than 60 bones recovered per taxa (Table 2; Fig 5D–5F). Worth mentioning are eight vertebrae assigned to a viper (probably the Palestine Viper cf. *Daboia palaestinae*) (Fig 5F). The low frequency, almost nonexistence, of vipers remains—the only true venomous snake identified at Eynan—is somewhat surprising. The most important and widespread viperid in Israel is *Daboia palaestinae* [3]. It is a large-medium snake with compact and robust vertebrae that should have had a better preservation rate than most of the vertebrae of medium and small snakes that have a higher number of remains. Therefore, the rarity of the viper’s remains is unrelated to the preservation and is probably genuine.

The rare snakes show a complex picture in relation to burning evidence. On the basis of burning marks alone the remains could be further divided into two: those with relatively high percentage of burning marks and those with almost none. Two species, the Asian Racer *Hemorrhois nummifer* (Fig 5D), and the *Natrix* sp. (Fig 5E), with only 28 bones retrieved for each, have 35.71% and 39% of burnt bones, respectively. This percentage of burning is higher than most of the large “colubrine” snakes. In contrast, all other rare species have 0 to 10% of burning. The viper cf. *Daboia palaestinae*, for example, with eight vertebrae, has none with burning marks. A possible explanation for this pattern could be a different distribution of the remains within the area studied. In order to test this, we compared the spatial distribution of the burnt bones of *H*. *nummifer* and *Natrix* sp., to the spatial distribution of large “colubrines” burnt bones and to the distribution of all burnt snakes remains, but could not find a pattern (S1 Fig).

**Fig 8:**
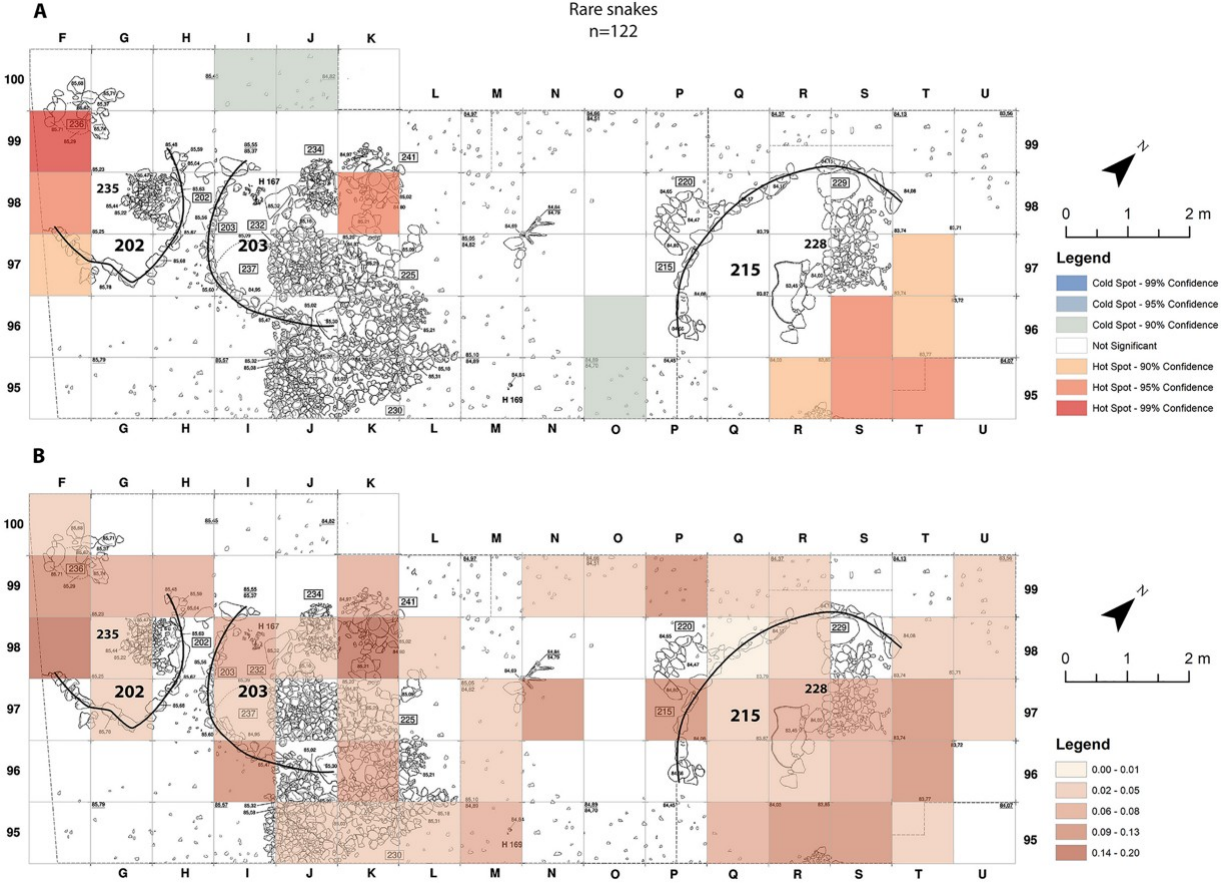
Rare snakes bone-distribution maps from Final Natufian Eynan (Ain Mallaha) with indication of the major buildings (202, 203 and 215). Rare taxa include: *Hemorrhois nummifer*, *Psammophis* cf. *schokari*, *Natrix* sp., *Eryx* sp. and cf. *Daboia palaestinae*. (A) “Hot-Spots Analysis Map” using Getis-Ord Gi statistic. (B) Natural Jenks distribution map based on the average sum of rare snake bones per 1 cm depth for each grid-square. The maps were created based on the modification of Eynan’s excavation map (Fig 1) and the bones grid-square location (https://doi.org/10.17026/dans-2c3-mn7q) using ESRI ArcMap 10.7.1.

The spatial distribution of the rare snakes is somewhat similar to that of the anurans and lizards (Fig 8). However, the hot-spot in the northern area is at the boundary between the inside and outside of building 215 (Fig 8A). An additional hot-spot with 95% confidence appears in square K98 (Fig 8A). This hot-spot is the result of the recovery of eight cf. *D*. *palaestinae* vertebrae within this square. The size, appearance, and spatial distribution of the vertebrae, indicate that they all belong to one individual (Fig 5F).

## Discussion

### Predators activities and natural death

The appearance of anurans and squamates at prehistoric archaeological sites is usually the result of predator activities or natural death at the site, especially in the case of trogloxene species in caves and shelters [49], and to a much lesser extent in open-air sites [45]. In the shelter of caves and at cave entrances toads, legless lizards and snakes can be found sometimes during excavations, since they use the cave and the loose sediment during hot summers or cold winters. Another contributing factor to cave accumulations are birds of prey that nest in caves, such as the Eagle-Owl (*Bubo bubo*) [36].

Recently, two taphonomic studies describing snake remains found in owl pellets were published [12, 36]. These join studies published in the last decade of snake remains from avian predation [33–35]. A remarkable outcome of the different actualistic taphonomic studies of avian predators is the dissimilarity of the results, even when the same species was responsible for the accumulation of the snake bones. The taphonomic signature of the Egyptian Vulture (*Neophron percnopterus*) was characterized by complete snake remains without any degree of digestion according to one study [33], in contrast to a high degree of digestion on some remains according to another [35]. Two studies [12, 36] characterized the Eurasian Eagle-Owl (*Bubo bubo*) as showing low fragmentation rates but varied digestion marks. While Lev et al. [12] report scarce high digestion levels (1%) on the vertebrae with mostly light digestion intensity, Lebreton et al. [36] report that the degree of digestion on the vertebrae is vastly influenced by the snake species, the degree of maturity of the prey, and the degree of mineralization of the vertebrae. The percentage of digested vertebrae, according to this study, is high and with heavy digestion for two snake species (*Zamenis scalaris* and *Natrix maura*). However, Lebreton et al. [36] also classify the digestion marks for a third snake taxon, *Coronella girondica*, as mainly light or moderate and rarely heavy. We must emphasize here that the two *B*. *bubo* studies used different categories to describe the digestion intensity: none, light, moderate, strong and heavy for one; none, light, moderate and high for the other. Their definition of each category and the areas of the vertebra affected were also dissimilar.

At the present state of research, caution is needed when interpreting snake remains on the basis of actualistic taphonomic studies if the same predator can create different taphonomic signatures. Another parameter to consider besides the preyed snake taxon and maturity, is the level of hunger of the predator that was reported to affect the variations in degrees of modification (p. 239 and references therein in [29]). Additionally, standardization of the indicators for the digestion intensity on snake vertebrae is needed, so that everyone will refer to the same intensity criteria affecting the same locations over the vertebrae. Furthermore, we currently have data only for few taxa of avian predators, but more avian species and many mammalian predators could be responsible for snake accumulations, each predator probably with a different taphonomic signature. Scats of mammalian predators occasionally feeding on snakes could have been present at a Natufian open-air site even while occupied. For example, foxes are abundant at Final Natufian Eynan and were reported as a species that coexisted with humans at the site while occupied [18]; dogs were also present and even buried with humans at the Early Natufian settlement of Eynan [50].

Eynan Final Natufian open-air settlement was intensively occupied by hunter-gatherers 12,000 years ago. During a site’s occupation, we expect reduced predator activity contributing to the accumulation of anurans and squamates within the settlement. Nonetheless, the natural occurrence of some animals within an open-air site where they can share space with humans is possible. The Roughtail Rock Agama *Stellagama stellio* is such an example since this heliotherm (sun-dependent) lizard was probably basking on the numerous built stone walls at Eynan even while it was occupied. In contrast, the occurrence of numerous very large “colubrines” within a settlement and inside dwellings during its occupation is more improbable. Therefore, we suggest that the accumulation of those large snakes was a result of human activities.

### Who was exploited?

Thousands of remains belonging to very large “colubrines”, exceeding 2 meters and sometimes even reaching roughly 3 meters long, were recovered in open areas, as well as in dwellings and other buildings (Fig 6). They seem to have been all over the site, a fact that reinforces the importance of their widespread exploitation within the settlement. This is even more clearly highlighted when compared with the frogs, toads, lizards, and rare snakes that were recovered, such as the viper. The high percentage of burning marks—on a third of all large “colubrines” remains—also emphasize that the accumulation of the bones was simultaneous with the occupation and not subsequent to it. It is possible to claim that the bones may have been disposed off in the sediment prior to the occupation and burnt later on when fireplaces were lighted. However, the massive number of large “colubrine” remains does not support such an assumption.

Snakes were gathered, carefully selected, and brought back to the settlement. Diurnal, large body length “colubrines” were picked by the Final Natufian habitants of Eynan from a wide range of snakes available at the vicinity of the site. Three species were considered appropriate, the Large Whip Snake *Dolichophis jugularis*, the Eastern Montpellier Snake *Malpolon insignitus* and an Eastern Four-lined Ratsnake *Elaphe* cf. *sauromates*, The Large Whip Snake *D*. *jugularis*, the most abundant of the three large “colubrines” identified, is also the longest of the Israeli snakes. It is a very impressive snake, completely shiny black in adulthood. Of the three species, it is also the most aggressive, defending itself by biting and raising its forebody off the ground. The Eastern Montpellier Snake *M*. *insignitus* is second only to *D*. *jugularis* in length, and in the number of remains recovered. It is thick, can seldom be aggressive, and although its bite can be severe since it is an opisthoglyphous snake, the grooved teeth are situated at the maxilla posterior extremity and therefore the venom can only be injected when the prey or an organ reach deep into its mouth. The Eastern Four-lined Ratsnake *Elaphe sauromates* is a thick snake that is an excellent climber. Nowadays it is found in Israel primarily at the very north, on Mount Hermon [3]. The possible presence of the Eastern Four-lined Ratsnake (cf. *Elaphe sauromates*) south of its current distribution during the Pleistocene, in the Hula Valley, was already evoked on the basis of a couple of vertebrae recovered at the prehistoric site of Nahal Mahanayeem Outlet [45].

Two other medium to large snakes, a water snake *Natrix* sp. (*Natricidae*) and the Asian Racer *Hemorrhois nummifer* (*Colubridae*), reaching up to 110 cm (the local *N*. *tesselleta*) and 170 cm (*H*. *nummifer*) [3], may have been exploited as well. Despite their rarity at Eynan, the percentage of burning marks for both is very similar to the large “colubrines” that were exploited. These were probably the two most commonly encountered snakes within the vicinity of the site. The Dice Snake *N*. *tessellata* is a very widely dispersed water snake in the Hula Valley nowadays, and a *Natrix* sp. was the most abundant snake recovered from other prehistoric archaeological sites in the area [45, 51]. The Asian Racer is one of the most common and most often encountered snakes in Israel nowadays [3]. Therefore, we suspect that these snakes may have been gathered ad-hoc when accidentally encountered, and considering the few remains attributed to them—less than 30 bones each—their contribution to the assemblage was not significant.

The only lizards that are suspected as having been exploited are the Roughtail Rock Agama lizards (*S*. cf. *stellio*), with an MNI of 18. However, this lizard is prevalent even today on stone walls in populated settlements and was probably widespread and easily spotted in the Final Natufian settlement on the numerous stone walls of the different buildings, even while occupied. Therefore if indeed exploited, it was opportunistically, like *Natrix* sp. and *H*. *nummifer*.

### Is it an expansion of the dietary breadth?

Snakes and lizards from Eynan were studied while bearing in mind that they were reported as part of the Natufian diet at many sites [8–12]. At Eynan, Bouchud [10] already noticed the abundance of large “colubrines” within "houses" and therefore suggested that they were consumed. More than 50 years ago, Bar-Yosef and Tchernov (p.133 in [8]), when reporting on the presence of reptiles at Hayonim Cave Natufian layer stated that:” Both [*Testudo graeca* and *Agama stellio* (actually *Stellagama stellio*)] were probably brought in as food by man, as were the snakes in all probability”. At El Wad Terrace Valla et al. (p.36 in [9]) concluded that "Snakes are common and, as much as *Ophisaurus*, were probably part of the people’s diet". Ever since, the consumption of *Pseudopus apodus* (previously *Ophisaurus*) alongside snakes, was repeatedly reported for the Natufian layers at El Wad Terrace as part of the expansion of the diet breadth related to the Broad Spectrum Revolution (BSR) [12, 52]. The only snake species reported as part of the diet at El Wad Terrace is *D*. *jugularis* [12], one of the large “colubrines” that was exploited at Final Natufian Eynan as well. However, there is no evidence to the exploitation of *P*. *apodus* at Eynan; although the NISP is high, it is one of the rarest species at Eynan based on the MNI (Table 1) with only 8% of burning marks (see the discussion in the results section).

The term Broad Spectrum Revolution (BSR) [53] is extensively used in reference to Late Pleistocene, and especially to the final Pleistocene subsistence-strategies in the Near East. The increase in the incorporation of "lower-ranked" taxa into the diet, or of resource diversification and intensification, are inferred by some as the result of depletion of preferred large "higher-ranked" game species as a result of long-term anthropogenic pressure [54–56]. Nevertheless, BSR resource diversification was also interpreted as the result of plenty [57, 58].

Eynan is situated at an ecotone offering a wide and highly diverse array of abundant resources. Varied aquatic habitats were available to its inhabitants, including an artesian spring approximately 100 m from the settlement with a constant year-round temperature of 21°-22°, a spring-fed river 3 km long that flows into the Hula Lake, and the adjacent Hula swamps [59]. Moreover, the site is situated at the foot of steep hills with "forest-park" Mediterranean woodland and surrounded by more moderate slopes with rocky and dryland grass fields [19]. The location of the Natufian settlement was significant for hunter-gatherers who could exploit several diverse habitats within an exceptionally short distance.

All snakes are edible, and the largest are usually the ones preferred, especially in time of food shortage—as was the case in France during world war II—because of the higher fat content [60]. Snake meat contains approximately 93 calories per 100g of raw meat, varying according to the snake taxon [61]. However, at Eynan a variety of vegetation and animals were available, as attested by the floral and faunal remains recovered [18–21, 62], and therefore two questions must be asked: Was the choice of eating specifically large “colubrine” snakes necessary at Eynan as a protein or fat source? Should the exploitation of supplementary species at the end of the Pleistocene be considered as associated merely with dietary requirements and calorie intake?

### Additional whys and wherefores of snakes exploitation

Large snakes are consumed in many parts of the world [60], nevertheless, consumption of a snake’s meat is commonly tied in with medicinal or other spiritual purposes [63].

#### The use of snakes in zoo-therapy

A snake coiled around a staff is nowadays the symbols of medicine and was in the past the symbol of the Greek god Zeus Asklepios, god of medicine. According to the mythology, the god sometimes revealed himself in the form of a snake, as he did at the time of the plague in Rome in 292 BC [1, 64]. Deities related to healing powers are frequently associated with snakes in ancient cultures [65–69], however, snakes are associated since antiquity with medicinal therapy, and not only as god’s representation; they repeatedly constitute essential elements of the *materia medica* [61, 65, 67, 70].

The use of snakes in zoo-therapy is rare in present-day Israel, but it still occurs. The Palestine Saw-scaled Viper *Echis coloratus* skeleton is used to solve intimacy problems, burnt snakeskin is used to heal inguinal hernia, and powder made of the shed skin of snakes is used to cure eyesight problems and eye diseases [71, 72].

Snakes and snakes body parts: blood, meat, fat, bile, bones, venom, and shed skin, have extensive therapeutic role worldwide [61, 68, 73–75]. A particular snake species or snake organ often have different remedial uses. Snake bones, for instance, are used in Africa for backaches and as snakebites antidotes; in Latin America to prevent teething pain in children; in Asia as a pain killer, to strengthen muscles and bones but also to expel wind and to remove dampness; and in Europe, it is used for rheumatic pain [61, 75, 76]. On the other hand, diverse species or snake organs can be used to treat the same illnesses. For example rheumatism, rheumatic pain or rheumatoid arthritis are treated in Zimbabwe with black mamba head or tail [75]; in Italy with Four-lined Snake fat [76]; in Slovenia with snake bones placed on the joint; in Brazilian folk medicine with fat of diverse snakes; in India with cobra’s fat or blood or with Rattlesnake oil; in Chinese medicine with snake bone powder; and in the African-American hoodoo tradition with snakeskin powder [61].

Interestingly one of the three large “colubrines” exploited at Eynan is an Eastern Four-lined Ratsnake (*E*. cf. *sauromates*). Until two decades ago, *E*. *sauromates* was defined as a subspecies of *E*. *quatuorlineata* but then was separated on the basis of genetic divergence and morphological traits [77]. *E*. *quatuorlineata* is a prominent snake in Mediterranean ritual medicine from south-eastern Europe. It was considered sacred in ancient Greece and Rome and is also used nowadays in religious rituals in Italy [78]. *E*. *quatuorlineata*’s saliva was probably used in Asklepios shrines alongside saliva of other non-venomous serpents to heal lesions [64]. The pharmaceutical properties of *E*. *quatuorlineata*’s saliva, which contains growth factors related to stimulation of healing processes, were confirmed by Angeletti et al. [64]. In southern Italy, *E*. *quatuorlineata*’s fat is used even nowadays as traditional medicine [76].

#### The role of snakes in the symbolic world

Besides their role in healing, snake products can be exploited as artisanal products; for ornamental use [74, 79–81] or as amulets and as charms to ward off the evil spirit or for good luck [61, 75, 82]. Sporadic finds of snake bones in ritual associations were reported from several archaeological sites: in a Danish Middle Bronze Age grave (p. 129 in [83]), in the Iron Age Qalat al-Bahrain snake burials [84] and in an Early Roman cistern in the Sanctuary of Poseidon [85].

Possible evidence to the importance of “colubrines” in the Natufian rituals may be found at Hilazon Tachtit Cave, a Late Natufian site in northern Israel [86]. The fill of an old woman’s grave interpreted as a “shaman” [87], and associated with funerary feast-remains and other offerings contained 1609 snake vertebrae identified as Colubridae vertebrae [88]. More data about the distribution of the snakes outside the “shaman’s” grave, the species represented, and an MNI, could contribute to a better understanding of whether the snakes were naturally present or an anthropogenic inclusion. Some researchers also refer to meander patterns on various Natufian objects as representing snakes [89]. Although this motif was present at Eynan on various stone items [13, 16], nonfigurative representations can have numerous meanings, and interpretation of these abstract motives as snakes is subjective.

In Northern Syria and Southeast Turkey, snakes played an important role in the symbolic world of early Pre-Pottery Neolithic (PPN) populations, which succeeded the Final Natufian of Eynan. Realistic figurative snakes were carved in bone and stone, or incised on stone vessels [90, 91]. One of the most impressive examples is Göbekli Tepe, where snakes are the animal most commonly depicted in reliefs on pillars in a ritual center [92]. However there are significant differences between the two cases, first in the PPN of Northern Syria and Southeast Turkey we are dealing with figurative snakes on objects, while at Eynan there is evidence of exploitation of snakes as expressed by abundant snake bones; second, the snakes depicted in the PPN are usually highly venomous vipers, whereas at Eynan venomous vipers are almost nonexistent in the faunal remains. Only eight vertebrae were recovered, and it seems vipers were intentionally avoided. There are those who would argue, however, that the scarcity or absence of a particular snake taxon in an archaeological site may hint to its special status in the society studied [81].

## Conclusions

We highlighted here the non-random exploitation of snake by the Final Natufian Eynan inhabitants. Diurnal large-bodied snakes were favored for exploitation. The anurans and squamates assemblage demonstrates that the inhabitant of the Natufian settlement did not catch all that was around them indiscriminately, but had clear preferences for large snake species, the largest amongst them, and non-venomous. Other snakes and lizards were less attractive, and anurans, including frogs, were probably not exploited. This conclusion could be drawn because of our ability to identify the remains to specific taxa. It emphasizes the importance of exhaustive taxonomic work prior to reaching any conclusion related to the exploitation of animals.

Moreover, in our view the exploitation was not due to shortage of food, since the snakes that were in all probability the most widespread in the vicinity of the site (*Natrix* sp. and *Hemorrhois nummifer*), were extremely rare in the assemblage. Only three carefully chosen species were targeted, from a variety of snakes available. We can therefore estimate that these three species had a special significance to the Final Natufian inhabitants of Eynan. Did they have a spiritual or mystical meaning? Were they known to have medicinal powers? Alternatively, maybe their skin was preferred for ornamentation, especially the shiny black skin of the *Dolichophis jugularis*. At the present state of research, we can only speculate. Hopefully further evidence in the future will enable us to reject or support some of our suppositions.

## Supporting information

S1 Fig Burnt snakes remains “Hot-Spots Analysis Map” using Getis-Ord Gi statistic from Final Natufian Eynan (Layer Ib). (A) *Natrix* sp.; (B) *Hemorrhois nummifer*; (C) large “colubrines”; (D) all snakes.
(PDF)

S1 Table Comparative collection specimens housed at the National Natural History Collections at the Hebrew University of Jerusalem, Israel (NNHC-HUJ). The number and centrum length of trunk vertebrae (post cervical) of comparative specimens of *Dolichophis jugularis* and *Malpolon insignitus*.
(PDF)

S2 Table Anurans and squamates taxa from Final Natufian Eynan (Layer Ib) Graves. Number of Identified Specimens (NISP).
(PDF)

S3 Table Snake vertebrae alterations from Final Natufian Eynan (Layer Ib), scoring by taxa. Categories after Lebreton et al. (2020): (0), non digested/corroded; (1) at least one of the diapophysis or parapophysis affected; (2) the addition of prezygapophyseal processes to the previous areas affected; (3) the addition of the condyle; (4) the addition of lamellar bone loss.
(PDF)
