# Supplementary material for: The anurans and squamates assemblage from Final Natufian Eynan (Ain Mallaha, Israel) with an emphasis on snake-human interactions
Source: PLoS One. 2021 Feb 25;16(2):e0247283. doi: 10.1371/journal.pone.0247283 (PMC7906325; doi:10.1371/journal.pone.0247283)
Supplement: S1 Fig — (A) Natrix sp.; (B) Hemorrhois nummifer; (C) large “colubrines”; (D) all snakes. (PDF) [file pone.0247283.s001.pdf]

S1 Figure

Burnt snakes remains “Hot-Spots Analysis Map” using Getis-Ord Gi statistic from Final Natufian Eynan (Layer Ib). A. *Natrix* sp.; B. *Hemorrhois nummifer*; C. Large “colubrines”; D. All snakes.

A.

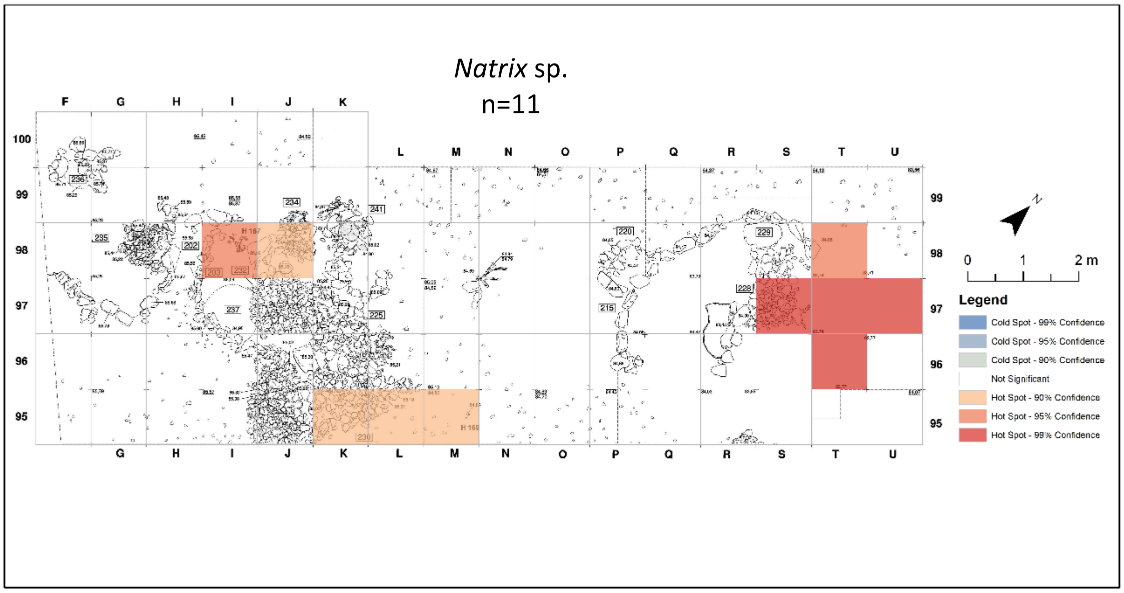

B.

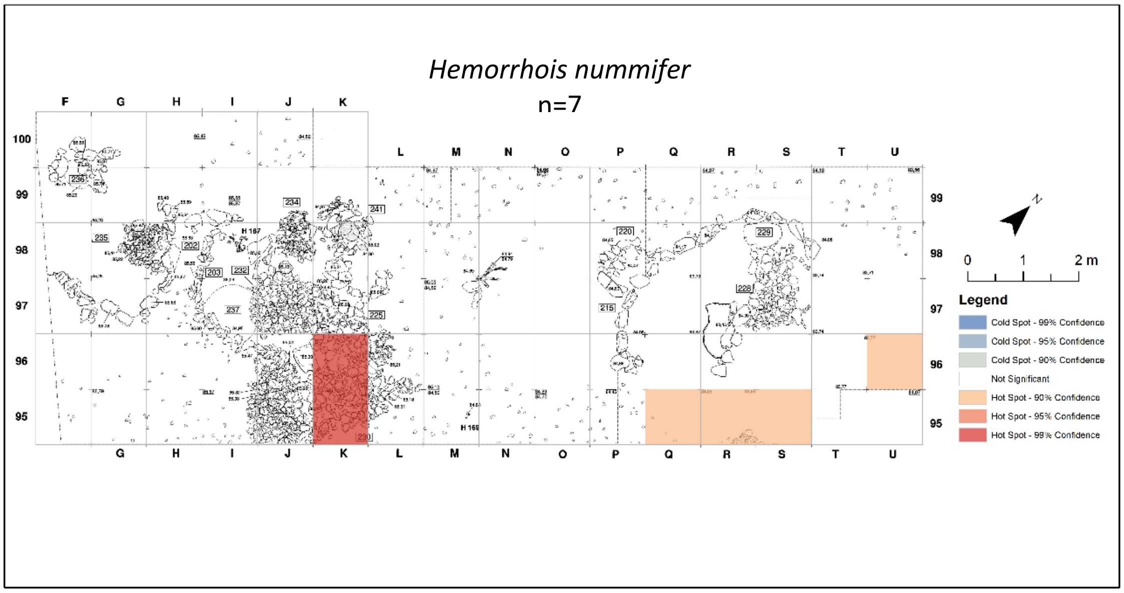

C.

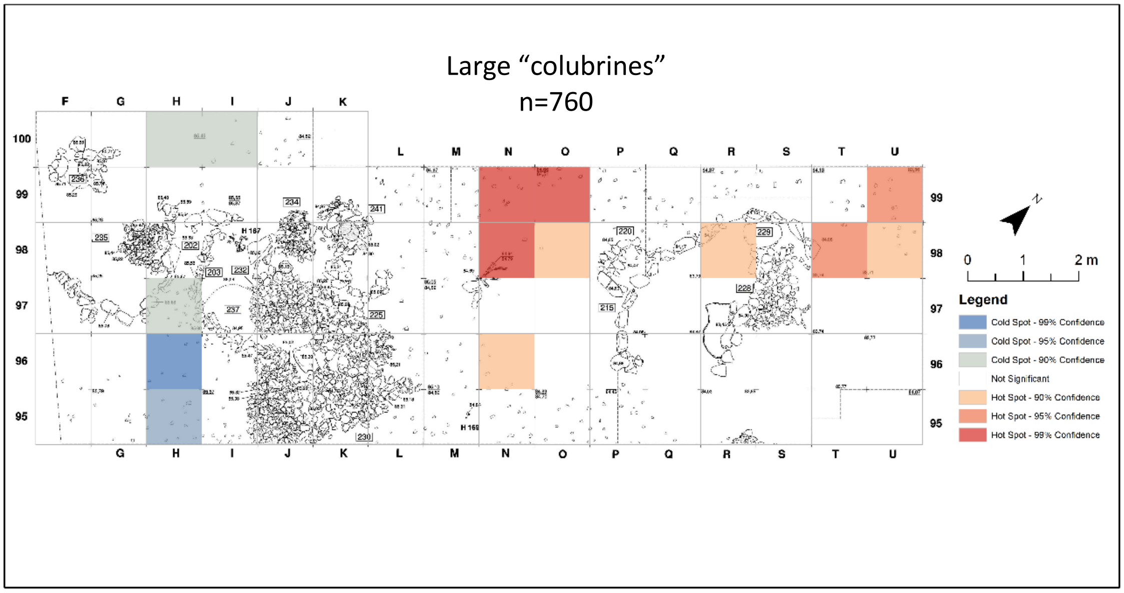
Large "colubrines"
n=760
F G H I J K L M N O P Q R S T U
100 99 98 97 96 95
0 1 2 m
Legend
Cold Spot - 99% Confidence
Cold Spot - 95% Confidence
Cold Spot - 90% Confidence
Not Significant
Hot Spot - 90% Confidence
Hot Spot - 95% Confidence
Hot Spot - 99% Confidence

D.

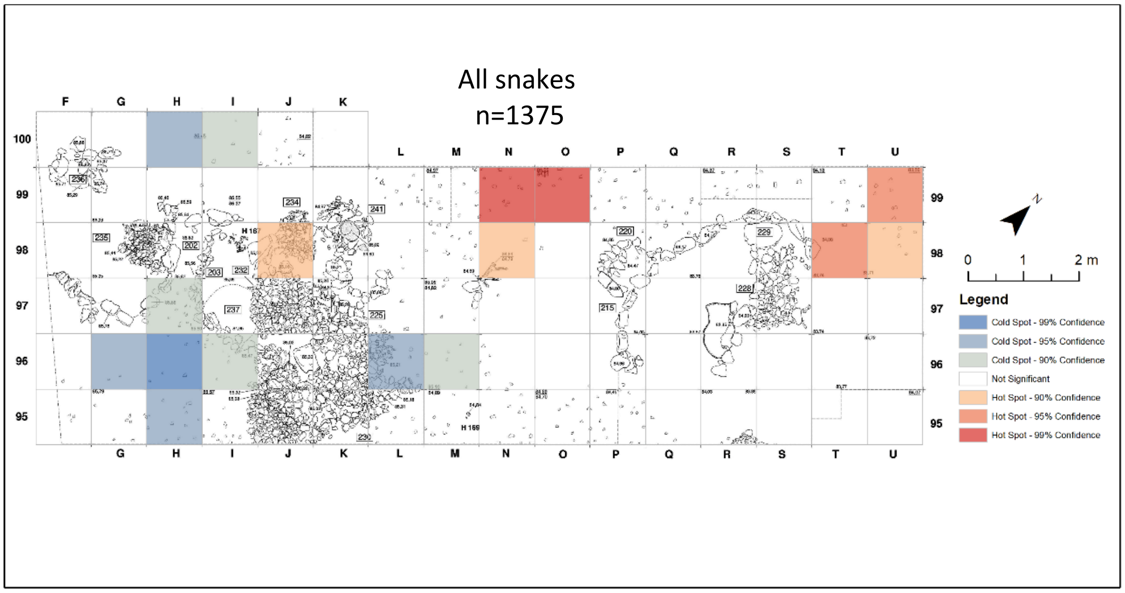
All snakes
n=1375
F G H I J K L M N O P Q R S T U
100 99 98 97 96 95
0 1 2 m
Legend
Cold Spot - 99% Confidence
Cold Spot - 95% Confidence
Cold Spot - 90% Confidence
Not Significant
Hot Spot - 90% Confidence
Hot Spot - 95% Confidence
Hot Spot - 99% Confidence
