# Supplementary material for: The anurans and squamates assemblage from Final Natufian Eynan (Ain Mallaha, Israel) with an emphasis on snake-human interactions
Source: PLoS One. 2021 Feb 25;16(2):e0247283. doi: 10.1371/journal.pone.0247283 (PMC7906325; doi:10.1371/journal.pone.0247283)
Supplement: S1 Table — The number and centrum length of trunk vertebrae (post cervical) of comparative specimens of Dolichophis jugularis and Malpolon insignitus. (PDF) [file pone.0247283.s002.pdf]

S1 Table

Comparative collection specimens housed at the National Natural History Collections at the Hebrew University of Jerusalem, Israel (NNHC-HUJ). The number and centrum length of trunk vertebrae (post cervical) of comparative specimens of *Dolichophis jugularis* and *Malpolon insignitus*.

Species	Specimen number NNHC-HUJ-Z-	Total length of specimen before skeletonized (cm)	Number of trunk vertebrae	Minimum length of trunk vertebrae (mm)	Maximum length of trunk vertebrae (mm)
Eastern Montpellier Snake *Malpolon insignitus*	95		83*	7.4	8.3
	388		122	3.9	4.9
	441	117	123	4.7	5.9
	448		154	3.8	4.5
	453		135	6.3	8.3
Large Whip Snake *Dolichophis jugularis*	322		150	4.8	5.8
	343**	130	158	4.4	4.8
	384**	110	159	4.0	4.8
	390	136	167	4.2	5.3
	393	193	160	6.6	7.7
	451**	55	145	2.3	2.8
	469		151	3.6	4.2

- *incomplete specimen
- **juvenile
