# Supplementary material for: The anurans and squamates assemblage from Final Natufian Eynan (Ain Mallaha, Israel) with an emphasis on snake-human interactions
Source: PLoS One. 2021 Feb 25;16(2):e0247283. doi: 10.1371/journal.pone.0247283 (PMC7906325; doi:10.1371/journal.pone.0247283)
Supplement: S2 Table — Number of Identified Specimens (NISP). (PDF) [file pone.0247283.s003.pdf]

S2 Table

Anurans and squamates taxa from Final Natufian Eynan (Layer Ib) Graves: Number of Identified Specimens (NISP) by graves (H).

	H150	H151-4	H156	H157	H158	H166	H167	H168	H170	Total
Anura indet.								1		1
Stellagama cf. *stellio*			1					1		2
Pseudopus apodus								3		3
Lizard indet.									1	1
Dolichophis jugularis	1		1	2				3		7
Malpolon insignitus			1					5	1	7
Elaphe cf. *sauromates*		1	2	2	3			3	1	12
Large "colubrine" indet.	2	2	7	5	2	1	5	15	7	46
Psammophis cf. *schokari*		1								1
"Colubriine" indet.			1	2				4		7
Eryx sp.								1		1
Snake indet.	6	1	12	10		10	6	38	10	93
Total	9	5	25	21	5	11	11	74	20	180
