# Supplementary material for: The anurans and squamates assemblage from Final Natufian Eynan (Ain Mallaha, Israel) with an emphasis on snake-human interactions
Source: PLoS One. 2021 Feb 25;16(2):e0247283. doi: 10.1371/journal.pone.0247283 (PMC7906325; doi:10.1371/journal.pone.0247283)
Supplement: S3 Table — Categories after Lebreton et al. (2020): (0), non digested/corroded; (1) at least one of the diapophysis or parapophysis affected; (2) the addition of prezygapophyseal processes to the previous areas affected; (3) the addition of the condyle; (4) the addition of lamellar bone loss. (PDF) [file pone.0247283.s004.pdf]

S3 Table

Snake vertebrae alterations from Final Natufian Eynan (Layer Ib), scoring by taxa. Categories after Lebreton et al. (2020): (0), non digested/corroded; (1) at least one of the diapophysis or parapophysis affected; (2) the addition of prezygapophyseal processes to the previous areas affected; (3) the addition of the condyle; (4) the addition of lamellar bone loss.

	Dolichophis jugularis	*Malpolon insignitus*	*Elaphe* cf. *sauromates*	Large “colubrine” indet.	*Hemorrhois nummifer*	*Psammophis* cf. *schokari*	“Colubrine” indet.	*Natrix* sp.	*Eryx* sp.	cf. *Daboia palaestinae*
n	356	205	119	1092	16	11	102	23	54	8
0	22.5%	20.5%	24.3%	10.6%	18.7%	18.2%	5.9%	0%	22%	0%
1	30.6%	28.8%	31.1%	20.7%	43.7%	45.5%	7.8%	22%	22%	12.5%
2	24.2%	25.4%	26.1%	19.5%	12.5%	18.2%	13.7%	44%	15%	37.5%
3	15.4%	10.7%	13.4%	13.5%	18.7%	9%	16.7%	17%	15%	37.5%
4	7.3%	14.6%	5.1%	35.6%	6.2%	9%	55.9%	17%	26%	12.5%
